# The development of the DISCO-RC for measuring children’s discomfort during research procedures

**DOI:** 10.1186/s12887-017-0949-y

**Published:** 2017-11-29

**Authors:** Mira S. Staphorst, Reinier Timman, Jan Passchier, Jan J. V. Busschbach, Johannes B. van Goudoever, Joke A. M. Hunfeld

**Affiliations:** 1 Department of Psychiatry, section of Medical Psychology and Psychotherapy, Erasmus University Medical Center, Room: Na-2013, PO box 2040, 3000 CA Rotterdam, The Netherlands; 2 Department of Clinical Psychology/EMGO+, VU University, Amsterdam, The Netherlands; 3 Department of Pediatrics, VU University Medical Centre, Amsterdam, The Netherlands; 4 Department of Pediatrics, Emma Children’s Hospital, Academic Medical Centre, Amsterdam, The Netherlands

**Keywords:** Adolescent, Child, Discomfort, Ethics committees, Questionnaire development, Research participation, Self report

## Abstract

**Background:**

There is a need for data on children’s self-reported discomfort in clinical research, helping ethics committees to make their evaluation of discomfort described in study protocols evidence-based. Since there is no appropriate instrument to measure children’s discomfort during medical research procedures, we aimed to develop a generic, short and child-friendly instrument: the DISCO-RC questionnaire (DISCOmfort in Research with Children).

**Methods:**

This article describes the six steps of the development of the DISCO-RC. First, we updated a literature search on children’s self-reported discomfort in clinical research to get insight in what words are used to measure discomfort (step 1). Subsequently, we interviewed 46 children (6–18 years) participating in research to get insight into important forms of discomfort for children (step 2), and asked them about their preferred response option for measuring discomfort (step 3). Next, we consulted nine paediatric research professionals from various backgrounds for input on the content and feasibility of the DISCO-RC (step 4). Based on the previous steps, we developed a draft version of the DISCO-RC, which we discussed with the professionals. The DISCO-RC was then pretested in 25 children to ensure face-validity from the child’s perspective and feasibility (step 5). Finally, validity, reliability and internal consistency were tested (step 6).

**Results:**

The search-update revealed several words used for measuring discomfort in research (e.g. ‘worries’, ‘unpleasantness’). The interviews gave insight into important forms of discomfort for children in research (e.g. ‘pain’, ‘boredom’). Children preferred a 5-point Likert scale as response option for the DISCO-RC. The experts recommended a short, digital instrument involving different forms of discomfort, and measuring discomfort of individual research procedures. Pretesting of the DISCO-RC resulted in a few layout changes, and feedback from the children confirmed the feasibility of the DISCO-RC. Convergent validity and test-retest reliability were acceptable. Internal consistency based on item-rest correlations and Cronbach’s alpha were low, as expected.

**Conclusions:**

The DISCO-RC is a generic, practical and psychometrically sound instrument for measuring children’s discomfort during research procedures. It contributes to make the evaluation of discomfort in paediatric research evidence-based. Therefore, we recommend including the DISCO-RC as standard component of paediatric research studies.

## Background

It is estimated that 25% to 65% of all drugs and treatment for children are prescribed off-label or unlicenced [1], which may put children’s health at risk to medication underdose or overdose. To improve paediatric health care, research is necessary [2]. However, paediatric research is only allowed under strict rules. In general, research is only acceptable when the Institutional Review Board (IRB) evaluated that the risks and discomfort are minimal, or that the benefits outweigh the risks and discomfort. Minimal risk and discomfort is when the probability and magnitude of harm or discomfort anticipated in the research are not greater than those ordinarily encountered in daily life or during the performance of routine physical or psychological examinations or tests [3]. Primarily in case of discomfort, IRBs base this evaluation on their intuition and experiences, which may not necessarily give a representative view of the actual experiences of the children [4–7]. Consequently, this can lead to the rejection of (parts of) studies when discomfort is expected to be excessive and, of course, vice versa. Preferably, the estimation of discomfort is based on group-level data of children’s discomfort during medical research procedures (i.e. medical procedures that are conducted for research purposes), but unfortunately these data are scarce. [8].

It is important to take children’s own perspectives into account when evaluating discomfort of research procedures to make this evaluation evidence-based. This argument is reflected in different reports, stating that it is necessary to define and permanently monitor children’s discomforts during research procedures [9, 10]. Moreover, it is reflected in Article 12 of the United Nations Convention on the Rights of the Child that children deserve to give their opinion in matters that concern them [11].

The need to have self-reported data about the experiences of children in clinical research is seen, for instance, by the development of the Reactions to Research Participation Questionnaire for Children (RRPQ-C) [12] and the Pediatric Research Participation Questionnaire (PRPQ) [13]. Although these questionnaires give a general view of paediatric research participation (e.g. trust in the research team), they give limited insight into discomfort, and do not address children’s experiences during the individual research procedures of a study. Since it is preferable that IRBs evaluate discomfort of the individual research procedures within a study [14, 15], the so-called component-analysis approach [16], it is important to have information on the discomfort of individual research procedures as well. Such information can be generalized across different research studies with similar procedures to estimate the level of discomfort that might be expected for children in future research with a given procedure.

In the absence of an appropriate instrument, we aimed to develop a questionnaire measuring children’s self-reported discomfort during medical research procedures. We aimed for a generic questionnaire that measures forms of discomfort relevant for all kinds of medical research procedures to enable comparisons between different research procedures, omitting aspects that are too specific (e.g. ‘feeling out of breath’ is only relevant for certain research procedures). We also aimed to use a very limited number of questions, as the questionnaire should be short and easy to complete, and we did not want our questionnaire to be an extra burden for the paediatric research participants. This paper describes the step-by-step development of the DISCO-RC questionnaire: DISCOmfort in Research with Children.

## Methods

### Step 1. Literature search

To gain insight into children’s discomfort in clinical research, two of the authors (JH and JP) first reviewed the state of knowledge regarding children’s discomfort or risk of children and adolescents who participate in research [8]. They searched literature from onset to December 2010. Inclusion criteria were: published in a peer-reviewed journal, empirical studies that addressed children’s self-reported experiences in clinical research, and written in English. Studies on parental burden, burden of illness, economic burden, non-empirical studies, and studies on the willingness to participate in medical research were excluded. They found eight articles concerning discomfort or risk. They concluded that studies on children’s self-reported discomfort in clinical research are scarce.

For the development of the DISCO-RC, MS and JH carried out an update and extension of this search (Appendix 1), for which PubMed, PsycINFO, Web-of-Science, Cochrane central, Medline and Embase were searched from onset to December 2012. They used the same inclusion criteria for the articles as the initial search. For item-generation, they looked at the main outcome measures described in the results section of the articles. In case of quantitative measures, these were often the topics of the questionnaires and in qualitative articles these were either the words mentioned by the children to describe their experiences or the themes that researchers identified from interviews.

### Step 2. Interview study

To incorporate the perspective of the children for item-generation, we conducted a qualitative study in 46 children (aged 6–18, M = 11.9, SD = 3.8) who participated in different kinds of research studies (experimental, observational or follow-up studies) at two paediatric academic hospitals. The aim of these interviews was to get insight into important forms of discomfort for children during research procedures, and what words they use to describe discomfort. A majority of these children (74%) was considered having a disease or medical condition (asthma, cystic fibrosis, cashew allergy, Inflammatory Bowel Syndrome) and an approximately even number of boys and girls was enrolled. Twenty-four different research procedures were performed on the children, which included both invasive (such as needle-related procedures, provocation tests) and non-invasive procedures (such as pulmonary function tests, taking medical history, questionnaires). Children were interviewed directly after the study visit. They were specifically asked to refer their participation related to the study visit that just ended to avoid recall-bias. The interview schedule is provided in Appendix 2.

Audiotaped interviews were transcribed verbatim and imported into NVivo 10.0 software [17]. To ensure anonymity, all identifying information was removed from the transcripts. Data were analysed using ‘thematic analysis’, which was chosen to categorise important themes related to discomfort [18]. The first author analysed the interviews and a supervising researcher independently analysed 25% of the interviews. Disagreements were discussed until consensus was reached.

### Step 3. Response option for the DISCO-RC

We searched for methods of the assessment of experiences of children in different developmental stages. Most researchers agree that in children from approximately 8 years and older questionnaires are a reliable way of measuring children’s experiences. However, there is no consensus in the literature what response option is best for children from 8 years onwards [19–24]. The aim of this step was therefore to ask children what type of response option they prefer for our questionnaire measuring discomfort. After the interview, children were asked to answer five written questions about their experiences with the research procedures. Forty-one of the 46 children (89%) of the previous step completed all five questions. These questions were based on input from literature, paediatricians, paediatric nurses and psychologists. Each question had three different types of response options: a 5-point Likert scale, a coloured numeric 100 mm visual analogue scale (VAS) (ranging from green ‘no discomfort’ to red ‘extreme discomfort’), and a simple 100 mm black line VAS. Children were asked to fill in all three response options for the five questions and at the end we asked them which option they preferred. We calculated Spearman correlations for the three different response options of each question to detect a possible discrepancy. We intentionally did not include a ‘faces’ scale because the reliability is often poor in older children [25], and we preferred to have a response option for children of all ages, without it coming across as being childish. Frequencies were used to determine children’s preference for a response option. We also determined whether there were preferences for a specific response option related to age differences by using a one-way ANOVA.

### Step 4. Consulting paediatric research professionals

The aim of this phase was to gain advice from an advisory panel of paediatric research professionals on the content, layout and the practical implementation of the questionnaire, based on the information that we had gathered from the previous steps. For this, we consulted nine paediatric research professionals from different backgrounds and disciplines: three paediatricians (one of them held a PhD in research ethics concerning paediatric research participation) with extensive experience in paediatric research; one paediatric research nurse; one paediatric research coordinator at an academic hospital (who was also a member of an IRB); a chairman of a parent association for children with Duchenne muscular dystrophy; two paediatric psychologists, and a pedagogue.

The information gained from the previous steps and the input from the professionals was used to develop a draft questionnaire of the DISCO-RC. We then presented this draft questionnaire to this group of professionals for additional review.

### Step 5. Pretesting the DISCO-RC

The draft version of the DISCO-RC was pretested on a diverse group of 25 healthy and ill children between 8 and 18 years old who participated in various clinical research studies in three academic paediatric hospitals to ensure feasibility and face-validity from the child’s perspective. The children were asked in a semi-structured interview by MS to comment on the content, the practical aspects and the form of the questionnaire. Questions included the relevance and comprehensiveness of the items, whether they understood the questions, whether using the questionnaire on an iPad-mini led to practical issues, and whether the time needed to complete the DISCO-RC was acceptable. Children were also asked if they had any other remarks on the questionnaire. The comments of the children were noted by the interviewer and discussed with the project team (i.e. authors of this article), leading to corresponding adaptations.

### Step 6. Psychometrics of the DISCO-RC

The final step of the development of the DISCO-RC was testing its psychometrics, using the scores of 418 children (8–18 years, M = 10.9 ± 2.1): 307 children in clinical research, 61 in routine clinical care (ultrasound imaging, MRI scans, pulmonary function test) and 50 children during dental check-ups. The latter two groups were included because of a research question for another study, but we also used them for validation purposes of the DISCO-RC. The minimum age for the child’s participation in this study was eight years, because the questionnaire we used for measuring convergent validity is only suitable for children aged eight and older (Children’s version of the Impact of Event Scale [26], and because some of the children aged six or seven (step 3) had difficulties with answering the written questions. An approximate equal percentage of boys and girls participated. About 75% of the children were healthy (i.e. they did not have a known disease). All children completed the DISCO-RC directly after undergoing the procedure.

#### Validity (convergent)

##### Event-related (traumatic) distress

For other research purposes, the Children’s version of the Impact of Event Scale (CRIES-13) was used [26]: a self-report scale that measures the frequency of event-related (traumatic) distress. This gave us the possibility to compare the scores of the DISCO-RC with those of the CRIES-13 as an indication of the convergent validity of the DISCO-RC. The CRIES-13 consists of 13 items, which are divided into three subscales: avoidance (four questions, e.g. ‘Did you try not to talk about it?’), intrusion/re-experiencing (four questions, e.g. ‘Did pictures about it pop into your mind?’), and arousal (five questions, e.g. ‘Did you get easily irritable?’). Children have to rate each question on a 4-point Likert scale, with the following weights and categories: 0 = ‘not at all’, 1 = ‘rarely’, 3 = ‘sometimes’, 5 = ‘often’. When a child has a total score of 30 or above on the CRIES-13, this child is considered to have clinically elevated stress response symptoms [27]. The CRIES-13 demonstrates satisfactory to good psychometric characteristics [28], and has good internal consistency for the total score (Cronbach’s *α* = 0.80). The CRIES-13 was administered to the children one month after their participation in research. They were specifically asked to rate the post-traumatic stress related to the research procedure on which they filled in the DISCO-RC. To measure the convergent validity of the DISCO-RC, we calculated a Spearman correlation between the average discomfort score of the DISCO-RC, which is based on the different forms of discomfort, and the total score of the CRIES-13. We expected that there would be a positive relation between the immediate discomfort of the children and later post-traumatic stress measured after one month, as we know from previous research that the subjective experience (e.g. peri-trauma fear and stress level) of a potentially intrusive event is a predictor/risk factor for post-traumatic stress. [29]

##### Parents’ ratings

We asked parents to rate their child’s annoyance during the procedures in order to measure convergent validity. We compared the children’s scores on annoyance (one of the questions of the DISCO-RC) with their parents’ ratings of annoyance by calculating the weighted kappa between these ratings [30, 31]. The reason for choosing ‘annoyance’ is that it reflects a general discomfort rather than a specific, and because we focused on children’s self-report rather than proxy reports.

#### Internal consistency

To evaluate the internal consistency of the DISCO-RC, we calculated Spearman correlations between each of the forms of discomfort (e.g. nervousness) and the discomfort score averaged across the other forms of discomfort while correcting for self-correlation (i.e. item-rest correlations). As discomfort was shown to be a multidimensional construct (Step 2), it was expected that these correlations would be modest, as well as the Cronbach’s alpha which was calculated too.

#### Test-retest reliability

We measured discomfort of the procedures twice: directly after undergoing the procedure and after one month. Test-retest reliability of the DISCO-RC was calculated with Spearman correlations of different forms of discomfort and the average discomfort score directly after the procedure and after one month. Additionally, to test the stability in level of discomfort over time, we analysed a possible difference between the two measurement moments with Wilcoxon’s signed rank tests.

## Results

**Fig. 1:**
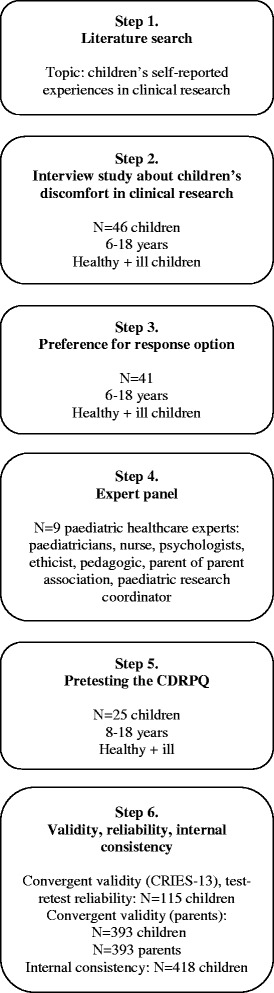
Scheme of the different steps of the development of the DISCO-RC

A diagram of the different steps of the questionnaire development is presented in Fig. 1.

### Step 1. Literature search

For the initial search 413 abstracts were identified of which eight were included that were focused on discomfort or risk. For item-generation of the DISCO-RC, we identified various potential items based on the included articles. Words that were used in quantitative articles as topics of the questionnaires were ‘discomfort’, ‘(un)comfortable’, ‘worried’, ‘pain’, ‘anxiety’ and ‘(un)pleasantness’. Words that children reported themselves to describe discomfort were ‘pain’, ‘worried’, ‘concerns’, ‘scared’ and ‘frightened’. The extension and update of the search from onset until December 2012 revealed 2780 potential articles, but did not reveal new original empirical studies on children’s self-reported discomfort related to medical research procedures in addition to the initial search.

### Step 2. Interview study

The majority of the children experienced various forms of discomfort related to the research procedures. Forms of discomfort that were frequently mentioned included feeling tired, having pain, feeling nervous/anxious because of anticipated pain or not knowing what to expect from the research study, shortness of breath, nausea, itchiness, and feeling hungry, feeling frightened, feeling bored because of the duration and/or waiting, and feeling ashamed. These various forms of discomfort suggest that discomfort is a multidimensional construct. We categorized these different forms of discomfort into two major themes across the procedures: physical and psychological/emotional discomfort. The extensive description of the results of the interview study is published elsewhere [32].

Half of the children, in particular the younger ones, did not know the meaning of ‘discomfort’ or ‘burden’ or did not know how to describe the word. The most frequently mentioned description by the children who did understand the word said discomfort means ‘annoying’.

### Step 3. Response option for the DISCO-RC

For each of the five questions, the three different response options (Likert scale, coloured numeric VAS, simple VAS) were strongly correlated (rho = 0.76–0.99, *p* < 0.01). Twenty-one children (51%) preferred the 5-point Likert scale, followed by 14 children (34%) preferring a coloured numeric 100 mm VAS (Table 1). Two children (aged six and eight) spontaneously said they would have preferred a faces scale. There were no age-related differences for the preferred response option (*p* = 0.21).

**Table 1:** Preference for response option

	Number of children	Percentage
5-point Likert scale	21	51.2%
Coloured 100 mm VAS	14	34.1%
Plain 100 mm VAS	2	4.9%
No preference	2	4.9%
5-point Likert scale or coloured 100 mm VAS	1	2.4%
Coloured 100 mm VAS or plain 100 mm VAS	1	2.4%
Total	41	100.0%

### Step 4. Consulting paediatric research professionals

During a group meeting, the paediatric research professionals gave practical and content-related suggestions for the development of the questionnaire. The most important suggestions are presented in Table 2.

**Table 2:** Suggestions of the paediatric research professionals about the questionnaire

Suggestions
1. The questionnaire should be administered **digitally** to make it more appealing to children and easier to distribute data to other researchers.
2. The questionnaire should be **short**, because children and parents are already loaded with questionnaires in research, e.g. about their health status. Questionnaires themselves are often perceived as a burden. They also indicated that the questionnaire should be short, so it does not interfere with their research studies.
3. **Parents** should also be asked to rate their child’s discomfort to study whether their ratings are similar.
4. The discomfort of **individual research procedures** should be measured because IRBs often evaluate the discomfort of the various research procedures of a study separately (i.e. component analysis approach).
5. Children do not like to fill in questionnaires that focus only on negative experiences. They should therefore also be asked about **positive experience** (i.e. whether children liked the research procedures).
6. As children are the subjects who undergo the procedures, they probably have good ideas how to improve these. Therefore, children should be asked about **improvements**, which is useful for researchers to minimize discomfort in their studies.
7. It is helpful to know whether children would undergo the research procedure in the **future** to get an impression of the child’s discomfort.

We presented the different forms of discomfort we gained from Step 1 (literature search) and Step 2 (interviews study with children) to the professionals, and discussed whether these should be relevant for our questionnaire. Based on Step 1, the forms of discomfort that were potentially suitable for our questionnaire were ‘pain’, ‘frightened’, and ‘worries’. These were the items that children mentioned themselves during interviews on open ended-questions. Items that we decided not to include were items that were made up by adults: ‘concerns’, ‘being (un)comfortable’, ‘aversion’, ‘anxiety’, ‘unpleasant’.

In consultation with the professionals, potential items based on Step 2 were: ‘feeling nervous’, ‘feeling annoyed’, ‘having pain’, ‘feeling frightened’, ‘feeling bored’, and ‘feeling tired’. Although ‘feeling ashamed/embarrassed’, ‘shortness of breath’, ‘nausea’, ‘itchiness’, and ‘feeling hungry’ are important forms of discomfort that were frequently mentioned by children as well, we decided not to include these as items, as these are only relevant for certain research procedures. The item ‘worries’ was deleted because it was mentioned in particularly by children during genetic susceptibility research and therefore might not be applicable to other sort of research.

Based on the previous steps and the advices from the professionals, we developed a draft version of the DISCO-RC. We discussed this draft version with the professionals until we reached consensus on the content and the phrasing of the questions. The items we decided to include in the questionnaire for pretesting in children were: ‘feeling nervous’, ‘feeling annoyed’, ‘having pain’, ‘feeling frightened’, ‘feeling bored’, and ‘feeling tired’.

In addition to the questions about discomfort, we followed the advice of the paediatric research professionals and added three questions to the DISCO-RC: 1) a question about ‘having fun’ so the questionnaire would not focus on negative experiences only, 2) a question about suggestions on to reduce discomfort related to the research procedure which can help researchers to improve their studies, and 3) a question whether children would undergo the research procedure in the future. We used Qualtrics© software to design a digital version of the DISCO-RC, which we used on an iPad-mini tablet.

### Step 5. Pretesting the DISCO-RC

All comments of the children regarding the content, practicability and feasibility of the questionnaire were noted. Children found it easy to complete the DISCO-RC and reported that they experienced no discomfort or burden because of the DISCO-RC. Instead, many said they liked filling in the DISCO-RC. They understood the questions and most considered the questions relevant for getting insight into their experiences during the research procedures. They said that they preferred an online questionnaire to a paper one, just as the expert panel had expected. However, the Internet connection failed sometimes (in three children), in which case the DISCO-RC was administered on paper. Some children considered the question about ‘liking the research procedure’ irrelevant. They said that if they did not like the research procedure, it did not mean that they experienced the procedure as discomforting, and vice versa. The children said that they did not mind that the questions were primarily about negative experiences. Furthermore, the children provided some recommendations to improve the layout of the questionnaire (i.e. larger font, fewer questions on one page).

With the input from the children in this step, we adapted the questionnaire to the preferences of the majority: we removed the question about liking the procedure as it was considered irrelevant by the children, and adapted the lay-out to the above-mentioned suggestions to make it easier to read. Although the Internet connection failed sometimes, we still decided to administer the questionnaire online (if possible), because children indicated that they prefer this way over a paper one. Also, online questionnaires might reduce social desirability bias.

### Step 6. Psychometrics of the DISCO-RC

#### Validity (convergent)

Although we aimed for asking both parents, there were no situations with both parents present. Usually it was the mother who filled in the proxy report. We observed a moderate Spearman correlation (*r* = 0.43; *p* < 0.001) between the average score on the DISCO-RC and the total score of the CRIES-13. The weighted kappa between the rating of the parents and children on the child’s annoyance was 0.41, which is considered moderate [33].

#### Internal consistency

Spearman correlations reflecting the contribution of the individual forms of discomfort on the average discomfort score are presented in Table 3. All the correlations though statistically significant (*p* < 0.05) were low, implying that discomfort is determined by diverse non-overlapping aspects. This was also illustrated by a low Cronbach’s alpha (0.547).

**Table 3:** Item-correlation with average discomfort score, test-retest correlations, differences in measurement moment

	Item-rest correlation with average score *N* = 418	*p*-value	Test-retest correlation *N* = 115	*p*-value^1)^	Wilcoxon’s Z N = 115	*p*-value
Nervousness	0.202	<0.001	0.665	<0.001	−0.011	0.991
Annoyed	0.401	<0.001	0.586	<0.001	−0.461	0.645
Pain	0.262	<0.001	0.725	<0.001	−0.600	0.549
Frightened	0.275	<0.001	0.525	<0.001	−0.209	0.835
Bored	0.163	0.001	0.440	<0.001	−1.213	0.222
Tired	0.248	<0.001	0.510	<0.001	−1.673	0.225
Average discomfort score	n/a	n/a	0.710	<0.001	−1.146	0.252

^1)^ *p*-values are one-sided

#### Test-retest reliability

The test-retest reliability of the items of the DISCO-RC, directly after the procedure and after one month, was high (Table 3). The retest scores did not differ significantly from the baseline scores for any of the items, although the reported discomfort was usually lower after one month.

The final version of the DISCO-RC is presented in Appendix 3 (Note: the DISCO-RC was developed in Dutch and then translated to English for this manuscript). We removed two questions from the final version, namely: ‘Did you like undergoing procedure X?’ and ‘Would you undergo research procedure X again in the future?’. The first question was removed because, like the pretest, a considerable number of children regarded this question as irrelevant; they said they did not mind the DISCO-RC focusing on negative experiences. The latter question was removed because on further consideration, it did not give additional insight into the child’s discomfort over and above the other questions.

## Discussion

This article describes the development of the DISCO-RC, which was designed to measure discomfort of common medical research procedures in children (8–18 years) in order to make the evaluation of discomfort in clinical research evidence-based. Since there is no ‘gold standard’ for measuring discomfort of research procedures (e.g. by self-report, by proxy, or by physiological measures such as cortisol levels), we focused on self-report because children’s self-reports are an important source of information that cannot be ignored and because we do not have much information on discomfort in research from children’s perspectives.

In general, we found that the DISCO-RC is a reliable and valid questionnaire to measure generic discomfort related to medical research procedures, and can be easily completed by children between the ages of 8 to 18. The children themselves indicated that they liked completing the DISCO-RC and did not experience it as burdensome.

The moment of measurement (directly after the procedures versus after one month) did not significantly influence children’s answers, although the reported discomfort was usually lower after one month. The discomfort we measured was low, making it difficult – if not impossible - to note (significant) differences in the reported discomfort after one month. We think for this reason it is necessary to validate the questionnaire during more invasive research procedures to study whether the level of discomfort changes over time. To avoid possible recall bias, we advise researchers to administer the questionnaire directly after the child underwent a research procedure.

Although the DISCO-RC was developed to measure discomfort in clinical research, the questionnaire could be used for measuring discomfort in clinical care as well. The reason why we focused on research is because there are strict guidelines for the level of discomfort, while these do not exist in clinical care. Although there are several instruments measuring children’s negative experiences in medical situations in clinical care [34–44], limitations of these instruments are that these primarily focus on the measurement of pain or distress. The interviews with the children showed that discomfort is an umbrella term that also represents other forms of discomfort than pain and anxiety. Measuring a variety of forms therefore provides a more thorough measure of the child’s discomfort in clinical research.

### Strengths and limitations

A strength of the DISCO-RC is that the content is based on literature, and input from children and paediatric healthcare professionals. It gives a good overview of discomfort experienced by children during research procedures in a short time. The DISCO-RC is a generic questionnaire that makes it possible to compare the discomfort caused by different research procedures. The DISCO-RC helps to identify discomfort from a procedures-related approach rather than a study-related approach, and therefore provides crucial complement to existing instruments measuring children’s experiences in research, such as the RRPQ-C and PRPQ. Furthermore, the DISCO-RC not only focuses on discomfort, but also on suggestions by children to reduce discomfort. This provides paediatric researchers with practical information to minimize discomfort of their studies, which is also a requirement of various ethical codes and regulations on paediatric research participation [3, 45].

We administered the DISCO-RC online, which has several advantages compared to paper-and-pencil questionnaires in terms of completeness of data (i.e. it can remind users that they skipped a question), less proneness to social desirability answering, and higher-cost effectiveness [46]. In addition, the outcomes of an online questionnaire can be easily stored online (anonymous obviously), which can make it easy for children, parents, IRBs, and paediatric researchers to have access to this information.

The DISCO-RC is limited in a way that for some procedures certain important forms of discomfort are not included, which may give an incomplete view of the overall discomfort. For instance, for children during Tanner staging, embarrassment may be an important for of discomfort. It is time-consuming to measure all forms of discomfort for all kinds of research procedures, which is why we decided to develop a generic questionnaire, suitable to compare discomfort of different research procedures and between different groups of children.

Also, the DISCO-RC primarily focuses on emotional discomfort: only two out of the six items of the questionnaire are related to physical discomfort (pain and tiredness). We think it would be more balanced when three items were related to physical and three items to emotional discomfort. Because no other generic physical discomfort was identified based on input from literature, the advisory panel and the children, we decided to stay with two items on physical discomfort.

The validation of the DISCO-RC is limited. For instance, measuring convergent validity between parents’ and children’s scores was only based on one of the questions of the DISCO-RC. Another limitation is the way ‘test-retest reliability’ is measured, which was based on retrospective recall. The situation during the retest is not equal to the first test, as time has passed since the research took place. A ‘real’ test-reliability would imply that the child would have to undergo the research procedure and the measurement of discomfort a second time, which is obviously unethical solely for the development of a questionnaire. However, assuming it has an equal effect on all children, the Spearman correlation we used is considered adequate for this purpose, as it reflects the order of the responses, not the level.

### Future research

Additional validation of the DISCO-RC is needed, as is validation in other languages. It would be helpful to measure convergent validity based on all questions of the DISCO-RC, reported by parents, researchers and children. Furthermore, future research is needed to investigate whether the DISCO-RC can also be used in younger children (< 8 years).

The discomfort we measured was low, making it difficult – if not impossible - to note differences in the reported discomfort after a month. We think for this reason it is necessary to validate the questionnaire during more invasive research procedures to study whether the level of discomfort changes over time.

### Future directions

The DISCO-RC can help to establish the level of discomfort of research procedures (i.e. ‘minimal’, ‘minor increase over minimal’, and ‘more than minimal’). As there is no clear description when a research procedure involves minimal discomfort, Westra et al. propose that “empirical data, expert opinions and/or the procedural characteristics suggest that at most a quarter of the persons (25%) concerned will experience considerable discomfort” [47]. Considerable discomfort could be conceptualized as children who reported “very” or “extremely” discomfort on the average score of the DISCO-RC.

To have information on children’s self-reported discomfort in research, it is necessary that these data are collected and disseminated. In order to collect information on children’s discomfort during research procedures, it would be helpful if paediatric researchers include the DISCO-RC as a standard component to their studies. We would advise to administer the questionnaire directly after the child underwent a certain research procedure. The information ideally would be published online (anonymously), so that the whole field of paediatric research can benefit from this information. IRBs can play a key role in this by requiring these data as part of a study protocol (Note: in the Netherlands it recently got obliged for paediatric researchers to describe the expected level of discomfort for the participating children) and recommending paediatric researchers to (centrally) register children’s experiences in an online repository.

## Conclusions

The DISCO-RC is a generic, short and practical instrument for measuring children’s discomfort during research procedures. It contributes to the evidence-based evaluation of discomfort in paediatric research. We recommend including the DISCO-RC as a standard component of paediatric research studies to measure children’s discomfort during medical research procedures.

## Appendix 1

### Search string to search for literature relating to children’s experiences in research

**EMBASE**

((annoy* OR anxiet* OR bored* OR emotion* OR fear* OR feeling* OR frustrat* OR helpless* OR irritat* OR mood* OR pleasure* OR regret* OR shame OR sorrow* OR coping OR cope OR coped OR stress* OR distress* OR burden OR perception* OR perceive* OR experience* OR comfort* OR discomfort*) NEAR/6 (procedure* OR technique* OR research* OR imaging* OR mri OR anesthe* OR anaesthe* OR intubat* OR surger* OR surgic* OR cannulat* OR infus* OR inject* OR ‘drug administration’ OR ‘x ray’ OR dialys* OR invasive OR noninvasive)):ab,ti AND (child/exp. OR newborn/exp. OR ‘child behavior’/de OR ‘child psychology’/de OR ‘child hospitalization’/de OR (infan* OR newborn* OR (new NEXT/1 born*) OR baby OR babies OR neonat* OR child* OR kid OR kids OR toddler* OR teen* OR boy* OR girl* OR minors OR underag* OR (under NEXT/1 ag*) OR juvenil* OR youth* OR kindergar* OR puber* OR pubescen* OR prepubescen* OR prepubert* OR pediatric* OR paediatric* OR school* OR preschool* OR highschool*):ab,ti OR ((adolescent/exp. OR adolescence/exp. OR adolescen*:ab,ti) NOT (adult/exp. OR aged/exp. OR ‘middle aged’/de OR (adult*):ab,ti))) AND (psychology/exp. OR ‘psychological aspect’/de OR (psychol*):ab,ti) AND (questionnaire/exp. OR ‘self report’/de OR interview/exp. OR ‘nonverbal communication’/exp. OR observation/de OR ‘clinical observation’/de OR (questionnaire* OR ((self OR child*) NEAR/3 report*) OR interview* OR nonverb* OR (non NEXT/1 verb*) OR observ*):ab,ti)

**Medline in OvidSP**

((annoy* OR anxiet* OR bored* OR emotion* OR fear* OR feeling* OR frustrat* OR helpless* OR irritat* OR mood* OR pleasure* OR regret* OR shame OR sorrow* OR coping OR cope OR coped OR stress* OR distress* OR burden OR perception* OR perceive* OR experience* OR comfort* OR discomfort*) ADJ6 (procedure* OR technique* OR research* OR imaging* OR mri OR anesthe* OR anaesthe* OR intubat* OR surger* OR surgic* OR cannulat* OR infus* OR inject* OR drug administration OR x ray OR dialys* OR invasive OR noninvasive)).ab,ti. AND (exp child/ OR exp. infant, newborn/ OR exp. child behavior/ OR child psychology/ OR (infan* OR newborn* OR (new ADJ born*) OR baby OR babies OR neonat* OR child* OR kid OR kids OR toddler* OR teen* OR boy* OR girl* OR minors OR underag* OR (under ADJ ag*) OR juvenil* OR youth* OR kindergar* OR puber* OR pubescen* OR prepubescen* OR prepubert* OR pediatric* OR paediatric* OR school* OR preschool* OR highschool*).ab,ti. OR ((adolescent/ OR adolescen*.ab,ti.) NOT (exp adult/ OR (adult*).ab,ti.))) AND (exp psychology/ OR (psychol*).xs,ab,ti.) AND (exp questionnaires/ OR Interviews as Topic/ OR Interview, Psychological/ OR exp. nonverbal communication/ OR observation/ OR (questionnaire* OR ((self OR child*) ADJ3 report*) OR interview* OR nonverb* OR (non ADJ verb*) OR observ*).ab,ti.)

**PsycINFO in OvidSP**

((annoy* OR anxiet* OR bored* OR emotion* OR fear* OR feeling* OR frustrat* OR helpless* OR irritat* OR mood* OR pleasure* OR regret* OR shame OR sorrow* OR coping OR cope OR coped OR stress* OR distress* OR burden OR perception* OR perceive* OR experience* OR comfort* OR discomfort*) ADJ6 (procedure* OR technique* OR research* OR imaging* OR mri OR anesthe* OR anaesthe* OR intubat* OR surger* OR surgic* OR cannulat* OR infus* OR inject* OR drug administration OR x ray OR dialys* OR invasive OR noninvasive)).id,ab,ti. AND (100.ag. OR exp. Child Attitudes/ OR child psychology/ OR (infan* OR newborn* OR new born* OR baby OR babies OR neonat* OR child* OR kid OR kids OR toddler* OR teen* OR boy* OR girl* OR minors OR underag* OR under ag* OR juvenil* OR youth* OR kindergar* OR puber* OR pubescen* OR prepubescen* OR prepubert* OR pediatric* OR paediatric* OR school* OR preschool* OR highschool*).id,ab,ti.) AND (exp psychology/ OR (psychol*).ab,ti.) AND (exp questionnaires/ OR exp. Interviews/ OR exp. nonverbal communication/ OR (questionnaire* OR ((self OR child*) ADJ3 report*) OR interview* OR nonverb* OR non verb* OR observ*).id,tm,ab,ti.)

**Cochrane central**

((annoy* OR anxiet* OR bored* OR emotion* OR fear* OR feeling* OR frustrat* OR helpless* OR irritat* OR mood* OR pleasure* OR regret* OR shame OR sorrow* OR coping OR cope OR coped OR stress* OR distress* OR burden OR perception* OR perceive* OR experience* OR comfort* OR discomfort*) NEAR/6 (procedure* OR technique* OR research* OR imaging* OR mri OR anesthe* OR anaesthe* OR intubat* OR surger* OR surgic* OR cannulat* OR infus* OR inject* OR ‘drug administration’ OR ‘x ray’ OR dialys* OR invasive OR noninvasive)):ab,ti AND ((infan* OR newborn* OR (new NEXT/1 born*) OR baby OR babies OR neonat* OR child* OR kid OR kids OR toddler* OR teen* OR boy* OR girl* OR minors OR underag* OR (under NEXT/1 ag*) OR juvenil* OR youth* OR kindergar* OR puber* OR pubescen* OR prepubescen* OR prepubert* OR pediatric* OR paediatric* OR school* OR preschool* OR highschool*):ab,ti OR ((adolescen*:ab,ti) NOT ((adult*):ab,ti))) AND ((psychol*):ab,ti) AND ((questionnaire* OR ((self OR child*) NEAR/3 report*) OR interview* OR nonverb* OR (non NEXT/1 verb*) OR observ*):ab,ti)

**Web-of-Science**

TS = (((annoy* OR anxiet* OR bored* OR emotion* OR fear* OR feeling* OR frustrat* OR helpless* OR irritat* OR mood* OR pleasure* OR regret* OR shame OR sorrow* OR coping OR cope OR coped OR stress* OR distress* OR burden OR perception* OR perceive* OR experience* OR comfort* OR discomfort*) NEAR/6 (procedure* OR technique* OR research* OR imaging* OR mri OR anesthe* OR anaesthe* OR intubat* OR surger* OR surgic* OR cannulat* OR infus* OR inject* OR “drug administration” OR “x ray” OR dialys* OR invasive OR noninvasive)) AND ((infan* OR newborn* OR (new born*) OR baby OR babies OR neonat* OR child* OR kid OR kids OR toddler* OR teen* OR boy* OR girl* OR minors OR underag* OR under age* OR juvenil* OR youth* OR kindergar* OR puber* OR pubescen* OR prepubescen* OR prepubert* OR pediatric* OR paediatric* OR school* OR preschool* OR highschool*) OR ((adolescen*) NOT ((adult*)))) AND ((psychol*)) AND ((questionnaire* OR ((self OR child*) NEAR/3 report*) OR interview* OR nonverb* OR (non verb*) OR observ*)))

**Pubmed publisher**

((annoy*[tiab] OR anxiet*[tiab] OR bored*[tiab] OR emotion*[tiab] OR fear*[tiab] OR feeling*[tiab] OR frustrat*[tiab] OR helpless*[tiab] OR irritat*[tiab] OR mood*[tiab] OR pleasure*[tiab] OR regret*[tiab] OR shame[tiab] OR sorrow*[tiab] OR coping[tiab] OR cope[tiab] OR coped[tiab] OR stress*[tiab] OR distress*[tiab] OR burden[tiab] OR perception*[tiab] OR perceive*[tiab] OR experience*[tiab] OR comfort*[tiab] OR discomfort*[tiab]) AND (procedure*[tiab] OR technique*[tiab] OR research*[tiab] OR imaging*[tiab] OR mri[tiab] OR anesthe*[tiab] OR anaesthe*[tiab] OR intubat*[tiab] OR surger*[tiab] OR surgic*[tiab] OR cannulat*[tiab] OR infus*[tiab] OR inject*[tiab] OR drug administration[tiab] OR x ray[tiab] OR dialys*[tiab] OR invasive[tiab] OR noninvasive[tiab])) AND ((infan*[tiab] OR newborn*[tiab] OR (new ADJ born*[tiab]) OR baby OR babies OR neonat*[tiab] OR child*[tiab] OR kid[tiab] OR kids[tiab] OR toddler*[tiab] OR teen*[tiab] OR boy*[tiab] OR girl*[tiab] OR minors[tiab] OR underag*[tiab] OR under ag*[tiab] OR juvenil*[tiab] OR youth*[tiab] OR kindergar*[tiab] OR puber*[tiab] OR pubescen*[tiab] OR prepubescen*[tiab] OR prepubert*[tiab] OR pediatric*[tiab] OR paediatric*[tiab] OR school*[tiab] OR preschool*[tiab] OR highschool*[tiab]) OR ((adolescen*[tiab]) NOT ((adult*[tiab])))) AND ((psychol*[tiab])) AND ((questionnaire*[tiab] OR self report*[tiab] OR child report*[tiab] OR interview*[tiab] OR nonverb*[tiab] OR non verb*[tiab] OR observ*[tiab])) AND publisher[sb]

## Appendix 2

### Interview schedule

*General experiences*

How did you feel about the study in general?

How did you feel before the study?

How did you feel afterwards?

Can you describe your experiences during the study?

Can you describe your experiences during procedure X?

*Experiences related to discomfort*

Can you describe any discomfort you experienced in the study?

Is there any part of the study that you did not like? Which part? Why?

Can you describe any discomfort you experienced because of procedure X?

*Worst experiences*

What was/were the most burdensome/discomforting part(s) of the study? Which part? Why?

*Preparation*

Who prepared you for the study?

What information did you get about the study? Was this information sufficient?Did you know what to expect of the study?

*Suggestions to reduce discomfort*

Can you think of anything that would have made the study easier for you? If so, could you tell me about it?

Can you think of anything that will make procedure X less discomforting for you? If so, could you tell me about it?

Can you think of anything that will make procedure X more comfortable for you? If so, could you tell me about it?

*Future research*

Would you participate in this research study again? Why (not)?

## Appendix 3

### DISCO-RC after evaluation

1. Were you nervous while undergoing procedure X?

□I was not nervous

□I was slightly nervous

□I was somewhat nervous

□I was very nervous

□I was extremely nervous

2. Was procedure X annoying?

□Procedure X was not annoying

□Procedure X was slightly annoying

□Procedure X was somewhat annoying

□Procedure X was very annoying

□Procedure X was extremely annoying

3. Was procedure X painful?

□Procedure X was not painful

□Procedure X was slightly painful

□Procedure X was somewhat painful

□Procedure X was very painful

□Procedure X was extremely painful

4. Were you frightened while undergoing procedure X?

□I was not frightened

□I was slightly frightened

□I was somewhat frightened

□I was very frightened

□I was extremely frightened

5. Were you bored while undergoing procedure X?

□I was not bored

□I was slightly bored

□I was somewhat bored

□I was very bored

□I was extremely bored

6. Did you find procedure X tiring?

□It was not tiring

□It was slightly tiring

□It was somewhat tiring

□It was very tiring

□It was extremely tiring

7. Do you have any suggestions for making procedure X less annoying?

Note: the DISCO-RC was developed in Dutch and translated in English for this article.

## Abbreviations

CRIES: Children’s version of the impact of event scale
DISCO-RC: DISCOmfort in research with children
IRB: Institutional review board
PRPQ: Pediatric research participation questionnaire
RRPQ-C: Reactions to research participation questionnaire for children
VAS: Visual analogue scale
